# A Systematic Review and Meta-Analysis on the Prognostic Value of BRCA Mutations, Homologous Recombination Gene Mutations, and Homologous Recombination Deficiencies in Cancer

**DOI:** 10.1155/2022/5830475

**Published:** 2022-07-20

**Authors:** Changxia Shao, Michael S. Chang, Fred C. Lam, Andrew R. Marley, Huilin Tang, Yiqing Song, Chelsey Miller, Madeline Brown, Isabella Wan, Jiali Han, Gboyega Adeboyeje

**Affiliations:** ^1^Merck & Co., Inc, Kenilworth, NJ, USA; ^2^Harvard Medical School, Boston, MA, USA; ^3^Division of Neurosurgery, Saint Elizabeth's Medical Center, Steward Medical Group, Brighton, MA, USA; ^4^Indiana University, Richard M. Fairbanks School of Public Health, Indianapolis, IN, USA; ^5^Integrative Precision Health LLC, Carmel, IN, USA

## Abstract

Patients with *BRCA1/2* mutations (*BRCA*m), loss-of-function mutations in other homologous recombination repair (HRRm) genes, or tumors that are homologous recombination deficiency positivity (HRD+) demonstrate a robust response to PARPi therapy. We conducted a systematic literature review and meta-analysis to evaluate the prognostic value of *BRCA*m, HRRm, and HRD+ on overall survival (OS) among those treated by chemotherapy or targeted therapy other than PARPi across tumor types. A total of 135 eligible studies were included. Breast cancer (BC) patients with *BRCA1/2*m had a similar overall survival (OS) to those with wild-type *BRCA1/2* (*BRCA1/2 *wt) across 18 studies. Ovarian cancer (OC) patients with *BRCA1/2*m had a significantly longer OS than those with *BRCA1/2 *wt across 24 studies reporting *BRCA1*m and *BRCA2*m, with an HR of 0.7 (0.6–0.8). Less OS data were reported for other tumors: 6 studies for *BRCA2*m compared with *BRCA2 *wt in prostate cancer with an HR of 1.9 (1.1–3.2) and 2 studies for *BRCA1/2*m compared with *BRCA1/2 *wt in pancreatic cancer with an HR of 1.5 (0.8–3.1). Only 4 studies reported HRD+ by either *BRCA *m or genomic instability score (GIS) ≥ 42 and OS by HRD status. The HR was 0.67 (0.43–1.02) for OS with HRD+ vs. HRD−. A total of 15 studies reported the association between HRRm and OS of cancers in which one or more HRR genes were examined. The HR was 1.0 (0.7–1.4) comparing patients with HRRm to those with HRR wild-type across tumors. Our findings are useful in improving the precision and efficacy of treatment selection in clinical oncology.

## 1. Introduction

Synthetic lethality arises when a combination of mutations in two genes leads to cell death, while mutation of either gene alone has no effect on cell viability [1]. The ability to create synthetic lethal relationships by pairing cancer-associated mutations with pharmacologic agents (at concentrations that would normally be nontoxic to healthy cells) has led to remarkable strides in cancer therapeutics [2]. The discovery that ovarian cancer cells harboring mutations in the homologous recombination repair (HRR) genes *BRCA1* and *BRCA2* exhibit synthetic lethality when treated with poly adenosine diphosphate (ADP)-ribose polymerase inhibitors (PARPi) has further expanded the application of PARPi in the clinic beyond *BRCA1/2* mutant cancers, with efforts to further identify genome-wide synthetic lethal vulnerabilities to this class of drugs [2]. This in turn led to the identification of other HRR gene mutations (HRRm) and characterization of homologous recombination deficient (HRD) cell state that renders cancer cells sensitive to PARPi [3, 4]. Current FDA-approved HRD biomarkers predicting response to PARPi include germline *BRCA* (*gBRCA*) mutations, platinum sensitivity as a surrogate biomarker for HRD, somatic mutations in HRR genes including *BRCA*, genomic scar HRD assays, and gene and protein expression profiling [5, 6].

The ability to broadly screen across cancer types using different biomarker assays to identify sensitivity to PARPi has produced clinical trials expanding their use as single-agent therapy or in combination with other DNA damage agents, targeted agents, or immunotherapies, across multiple tumor types. PARPi has demonstrated broad application in the treatment of cancer patients with *BRCA* mutations (*BRCA*m), HRRm, and HRD positivity. However, little is known regarding whether the presence of these genetic alterations alone affects overall survival (OS) in cancer patients not treated with PARPi or immunotherapy. We performed a systematic review and meta-analysis to examine the prognostic value of these biomarkers across multiple cancer types in predicting OS in cancer patients treated with chemotherapy or targeted therapy other than PARPi.

## 2. Methods

### 2.1. Study Design and Search Strategy

This study was performed as per the the Preferred Reporting Items for Systematic Reviews and Meta-Analysis (PRISMA) guidelines. Relevant studies with full-text articles in the last 10 years and conference abstracts in the last 3 years were identified by searching the following databases: Ovid Medical Literature Analysis and Retrieval System Online (MEDLINE), Excerpta Medica database (EMBASE), Cochrane Central Register of Controlled Trials, and Cochrane reviews. Searches were performed on May 21, 2020, using relevant terms in English. Two reviewers independently selected studies according to the inclusion criteria, with a third independent reviewer available to address any discrepancies. Bibliographies from review articles were reviewed thoroughly to identify relevant studies, ensuring that papers and articles not picked up in the original search were also included. Studies involving patients treated with chemotherapy or targeted therapy other than PARPi were included in the analysis.

HRD was defined as having either deleterious or suspected deleterious *BRCA1/2*m or a genomic instability score ≥42 by the Myriad testing (standard definition), with an alternative definition as only having a genomic instability score ≥42 [5, 7]. The genomic instability score is an algorithmic measurement of loss of heterozygosity, telomeric allelic imbalance, and large-scale state transitions using DNA isolated from formalin-fixed paraffin-embedded tumor tissue specimens. The Myriad myChoice® HRD assay was used in the relevant studies to obtain the genomic instability score.

The eligibility criteria are listed in Supplementary Table 1, with a focus on clinical outcomes, defined as OS across all cancers and subtypes harboring *BRCA1/2m* and *HRRm* (defined as mutations in one or more of the following genes: *ATM, BARD1, BRCA1, BRCA2, BRIP1, CDK12, CHEK1, CHEK2, FANCA, FANCL, PALB2, PPP2R2A, MRE11 A, NBN, RAD50, RAD51, RAD51 B, RAD51 C, RAD51D,* and *RAD54 L*), and HRD status.

### 2.2. Data Analysis

Hazard ratios (HRs) for OS with corresponding 95% confidence intervals (CIs) were calculated across patients with or without *BRCA1/2*m, HRRm, and HRD status. Cochrane's Q test and the *I*^2^ statistic were used to assess heterogeneity between studies, with a *P* value < 0.05 for Cochrane's Q test and *I*^2^ > 50% considered cutoffs for significant heterogeneity [8, 9]. Publication bias was assessed by contour-enhanced funnel plots of standard error against the effect estimate. We performed a meta-analysis by tumor type and mutation status using a random-effects model based on the degree of heterogeneity between individual studies and presented data as forest plots. All statistical analyses were performed using STATA (Version 14; Stata Corp., College Station, TX). For studies that presented Kaplan–Meier survival data without reporting HR, we used a previously published methodology for estimating HR from time-to-event analyses [10].

## 3. Results

### 3.1. Study Demographics

**Figure 1:**
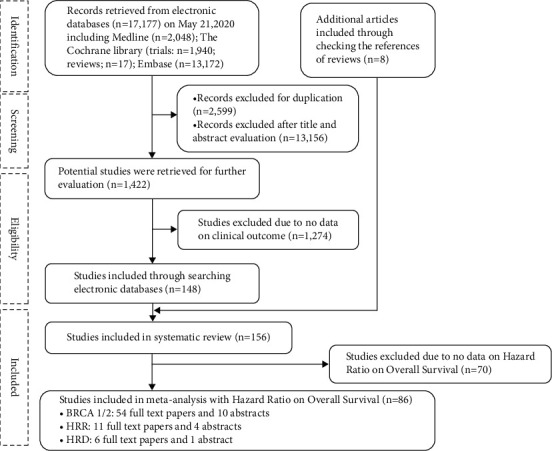
Flowchart of study selection.

Our PRISMA study protocol is shown schematically in Figure 1. We identified 86 outcomes studies on *BRCA1/2*m, HRRm, and HRD positivity and OS in our systematic review and meta-analysis across types of cancer. Citation lists are presented in Supplementary Tables 2, 3, and 4 for *BRCA1/2*m, HRRm, and HRD positivity, respectively.

### 3.2. Overall Survival and *BRCA1/2*m

**Figure 2:**
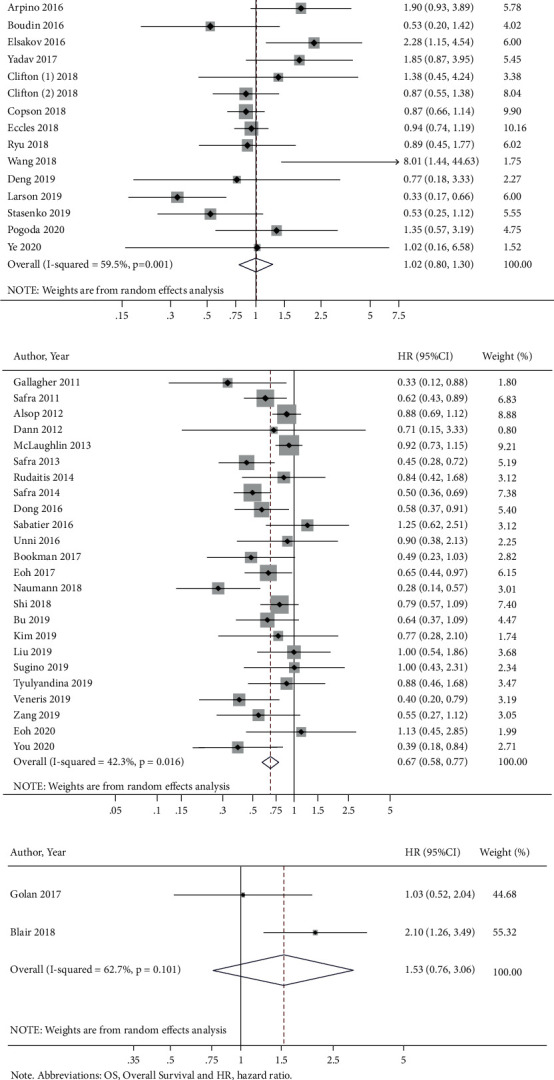
BRCA1/2 mutations and OS by cancer type. (a) Breast cancer. (b) Ovarian cancer. (c) Pancreatic cancer. Clifton (1), neoadjuvant group; Clifton (2), adjuvant group; OS, overall survival; and HR, hazard ratio.

We found no association between *BRCA1/2*m and OS among breast cancer patients (HR = 1.02 (95% CI = 0.80–1.30)) (Figure 2(a)). Furthermore, stratification revealed no differences in OS in patients with germline *BRCA1/2*m (Supplementary Figure 1(a)) and in patients with pathogenic variants (HR = 1.30 (95% CI = 0.93–1.81)) (Supplementary Figure 1(b)). Similarly, there was no effect on OS in triple-negative breast cancer patients or subgroup analysis among patients with germline mutations status (HR = 1.10 (95% CI = 0.75–1.60)) (Supplementary Figure 1(c)) or pathogenic variants (HR = 1.38 (95% CI = 0.45–4.19)) (Supplementary Figure 1(d)).

Compared to ovarian cancer patients with tumors that were *BRCA1/2* wt, ovarian cancer patients with tumors harboring *BRCA1/2*m had a better OS (HR = 0.67 (95% CI = 0.58–0.77)) (Figure 2(b)). Similar results were found among patients harboring germline mutations compared with those wild-type patients (HR = 0.69 (95% CI = 0.59–0.81)) (Supplementary Figure 1(e)). Patients with tumor somatic mutations did not have significantly different OS compared with those with wild-type *BRCA1*/2 (HR = 0.67 (95% CI = 0.23–1.93)). Given only two studies in this subgroup analysis, the results should be interpreted with caution (Supplementary Figure 1(f)). Similarly, stage III or IV ovarian cancer patients with tumors that were *BRCA1/2*m had significantly better OS than patients with *BRCA1/2* wt tumors of the same stage (HR = 0.64 (95% CI = 0.55–0.75)) (Supplementary Figure 1(g)). These results should also be interpreted with caution since many studies may not have fully documented the patient treatment history. In addition, the retrospective nature of the studies included in this analysis could be susceptible to selection bias, other potential biases, or confounding.

Few studies evaluating OS and *BRCA1*/*2*m for other cancer types were found. Our analysis of pancreatic cancer patients did not detect any effects of *BRCA1/2*m on OS (Figure 2(c)). Taken together, these results display discordant behavior of *BRCA1/2*m between patients with breast or ovarian cancer, suggesting possible tumor-intrinsic properties of ovarian cancers that combine with the presence of *BRCA1/2*m to lead to longer survival.

### 3.3. Overall Survival and *BRCA1*m

**Figure 3:**
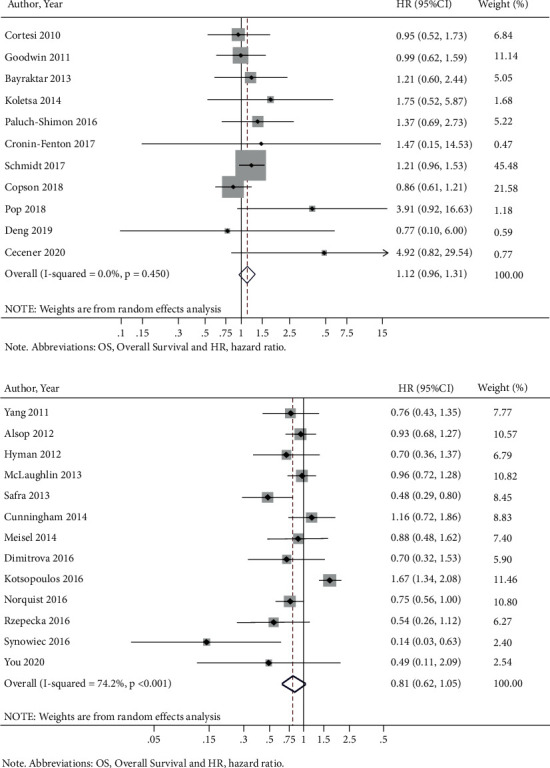
BRCA1 mutations and OS by cancer type. (a) Breast cancer. (b) Ovarian cancer.

Similar to what we found in breast cancer patients with *BRCA1/2*m, we found no significant association in OS in such patients harboring only *BRCA1*m (HR = 1.12 (95% CI = 0.96–1.13)) (Figure 3(a)), regardless of somatic or germline origin (Supplementary Figures 2(a) and 2(b), respectively), pathogenic mutational status (Supplementary Figure 2(c)), or triple-negative receptor status (Supplementary Figure 2(d)). *BRCA1*m alone was not significantly associated with an altered OS among ovarian cancer patients (HR = 0.81 (95% CI = 0.62–1.05)) (Figure 3(b)), regardless of germline or somatic origin (Supplementary Figure 2(e) and 2(f)), pathogenicity (Supplementary Figure 2(g)), or tumor stage (Supplementary Figure 2(h)).

### 3.4. Overall Survival and *BRCA2*m

**Figure 4:**
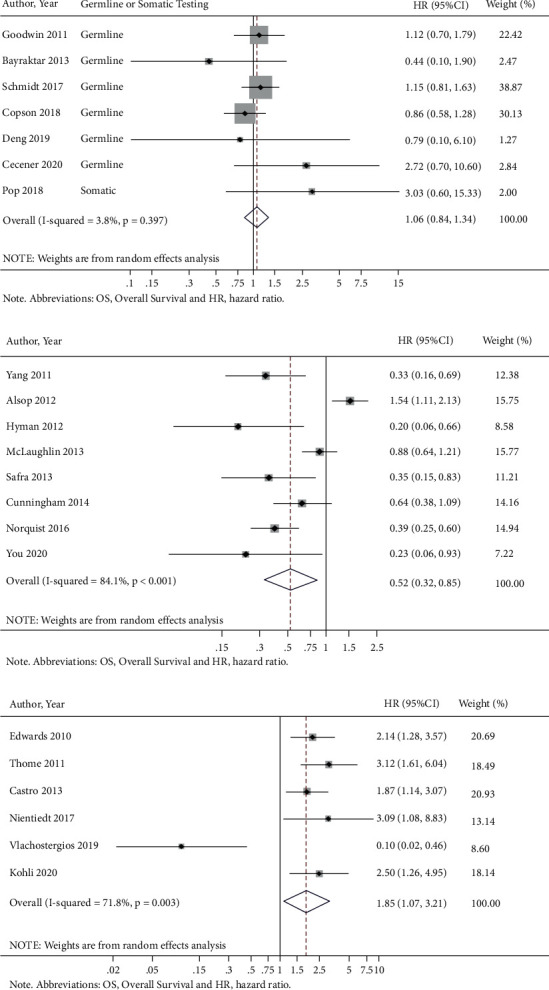
BRCA2 mutations and OS by cancer type. (a) Breast cancer. (b) Ovarian cancer. (c) Prostate cancer.

*BRCA2*m was not associated with OS in breast cancer patients (HR = 1.06 (95% CI = 0.84–1.34)) (Figure 4(a)) but was associated with improved OS in ovarian cancer patients (HR = 0.52 (95% CI = 0.32–0.85)) (Figure 4(b)). Subgroup analysis on germline and pathogenic *BRCA2*m did not show significantly different OS in breast cancer patients (Supplementary Figures 3(a) and 3(b), respectively) but was associated with longer OS in ovarian cancer patients (Supplementary Figure 3(c)–3(g)). Finally, we performed a meta-analysis comparing the effects of *BRCA2*m on OS in prostate cancer patients, which suggested that prostate patients with *BRCA2*m had worse OS than *BRCA2 *wt (HR: 1.85 (95% CI = 1.07–3.21)). Similar findings were observed for subgroup analyses by germline mutations or pathogenic mutations (Figure 4(c) and Supplementary Figures 3(h)–3(j)).

### 3.5. Overall Survival and HRRm

**Figure 5:**
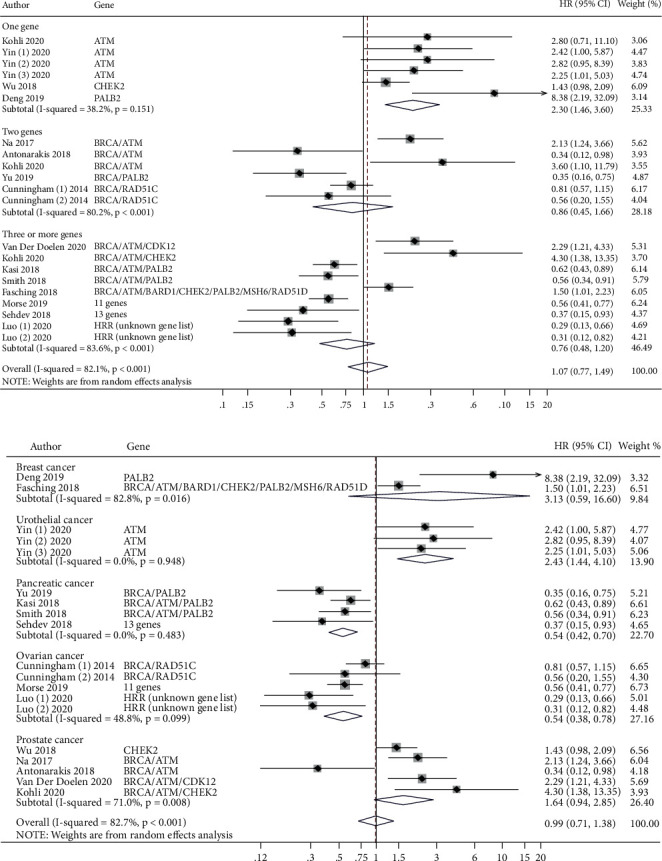
(a) HRR gene mutations and OS by the number of genes in each study. HR, hazard ratio; Yin (1), discovery set; Yin (2), validation set 1; Yin (3), validation set 2; Cunningham (1), germline mutations; Cummingham (2), somatic mutations; Luo (1), TCGA dataset; and Luo (2), ICGC dataset. (b) HRR gene mutations and OS by cancer type. HR, hazard ratio; Yin (1), discovery set; Yin (2), validation set 1; Yin (3), validation set 2; Cunningham (1), germline mutations; Cummingham (2), somatic mutations; Luo (1), TCGA dataset; and Luo (2), ICGC dataset.

We next performed a meta-analysis of studies reporting survival outcomes in patients with one, two, and three or more HRRm, as different gene lists and methodology were used across HRRm studies. There was no association between HRRm and OS (HR = 1.07 (95% CI = 0.77–1.49)) (Figure 5(a)). Interestingly, subgroup analysis from four studies demonstrated dramatically worse OS in patients with mutations in the DNA damage sensor kinase *ATM*, known to be essential in repairing damaged DNA and maintaining genome stability [11, 12] (HR = 2.47 (95% CI = 1.52–4.03)) (Supplementary Figure 4(a)). Further subgroup analyses of studies in patients with mutations in two HRR genes (*BRCA*/*PALB2*, *BRCA*/*RAD51 C*, or *BRCA*/*ATM*) (HR = 0.86 (95% CI = 0.45–1.66)) (Supplementary Figure 4(b)) or three or more HRRm (HR = 0.76 (95% CI = 0.48–1.20)) (Supplementary Figure 4(c)) did not demonstrate a significant effect on OS.

Meta-analyses did not demonstrate an association between HRRm and OS in cancer patients overall (HR = 0.99 (95% CI = 0.71–1.38)). An elevated risk of death was observed in patients with urothelial cancer harboring *ATM* mutations (HR = 2.43 (95% CI = 1.44–4.10)) (Figure 5(b)). Interestingly, patients with pancreatic and ovarian cancers harboring mutations in some of the HRR genes demonstrated a lower risk of death than patients without such mutations (HR = 0.54 (95% CI = 0.42–0.70) and HR = 0.54 (95% CI = 0.38–0.78), respectively) (Figure 5(b)).

### 3.6. Overall Survival and HRD Status

**Figure 6:**
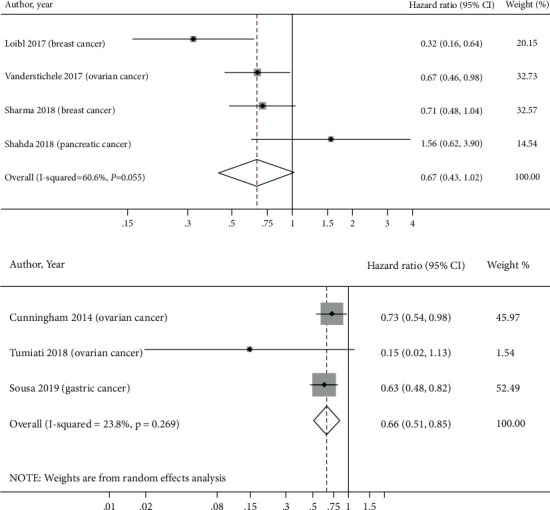
A HRD positivity (standard definition) and OS. A random-effects meta-analysis of 4 studies regarding the therapeutic effects comparing HRD high levels to low levels among all patients. Each square indicates the hazard ratio (HR) for overall survival (OS) in each study. The square size is proportional to the precision of HR (inverse of variance). The horizontal line represents the 95% confidence interval. Studies are ordered by the year of publication. The pooled HR and 95% CI are indicated by the dashed line and diamond, respectively; the black vertical line represents the null hypothesis.

Analysis of four studies in breast, ovarian, and pancreatic cancer patients suggested a trend of increased OS with HRD positivity (HR = 0.67 (95% CI = 0.43–1.02)) (Figure 6(a)). However, these results should be interpreted with caution. Similarly, we were only able to identify three studies that reported outcomes in patients with ovarian or gastric cancer using an alternate definition of HRD as a genomic instability score ≥42 regardless of *BRCA*m status, demonstrating an HR of 0.66 (95% CI = 0.51–0.85) (Figure 6(b)). Given the limited power of this meta-analysis, it is not possible to draw conclusions based on these results.

### 3.7. Publication Bias

In addition, the funnel plot showed no clear evidence of publication bias in any of these BRCA1/2, HRR, or HRD meta-analyses (data not shown).

## 4. Discussion

The prognostic value of pathogenic mutations in *BRCA1/2* and other HRR genes and HRD positivity is not fully understood in cancer patients not treated with PARPi. In this systematic review and meta-analysis, the presence of *BRCA1/2*m was significantly associated with better OS in ovarian cancer patients, but not in other cancer types. The results should be interpreted with caution due to the lack of full-treatment history and potential bias and confounding of included retrospective studies. For HRRm, no associations between HRRm and OS were observed across studies. In subgroup analyses, we observed a positive association between *ATM*m and urothelial cancer, but inverse associations between HRRm and pancreatic and ovarian cancers. These findings should be interpreted with caution due to a limited number of studies and studies with variable design and quality.

The UK prospective cohort (POSH) study assessed OS in 2,733 women below 40 years of age at first diagnosis with invasive breast cancer harboring *BRCA1/2*m. Those researchers found insignificant differences in survival, but triple-negative breast cancer patients harboring *BRCA1/2*m had a slight survival advantage in the first several years following their primary diagnosis [13]. A second study comparing 3,345 women with stages I–III breast cancer, 233 of who harbored a *BRCA1*m, also found a survival rate similar to that in women without that mutation, with improved survival following oophorectomy [14]. A third study in early onset triple-negative breast cancer patients assessing the presence of *BRCA*m and OS showed better outcomes, likely due to the increased response to anthracyclines and taxane-based chemotherapies [15]. Finally, a fourth study evaluating the outcomes of germline *BRCA1/2*m in patients with advanced high-grade serous ovarian cancer revealed longer progression-free survival compared to patients without germline mutations [16]. These studies echo our findings in both breast (Figure 2(a)) and ovarian (Figure 2(b)) cancer patients harboring germline *BRCA1/2*m, as well as in our analysis of breast and ovarian cancer patients with *BRCA1*m or *BRCA2*m alone, helping to validate our meta-analysis results across cancer types.

A recent study linking HRD scores in samples from patients with high-grade serious ovarian cancer (HGSOC) with clinical prognosis found that *BRCA1/2*m was more common in those patients' samples with HRD scores ≥63 and had a better prognosis compared to patients with HDR scores ≤62. HRD caused by gene alterations was associated with a better prognosis than HRD caused by epigenetic changes or unknown variant changes [17]. Similarly, another study used an HDR score cutoff of ≥33 (≥42 is currently used in the clinic) and found that this cutoff was associated with better OS in patients with epithelial ovarian cancer [18]. Analysis of HRRm other than *BRCA1/2*m in the TCGA project revealed that only patients with homozygous deletions in *CHEK1* and *PTEN* showed high HRD scores, but not patients with mutations in *ATM*, *ATR*, *FANCA*, *FANCD2*, *FANCM*, or *PALB2* [17]. Long-term survival in these patients depended on aggressive debulking of their primary disease, suggesting that patients with pathogenic HRRm should undergo surgical resection. Similarly, our meta-analysis also showed that patients with mutations in HRR genes did not have worse OS (Figure 5(b)), and HRD positivity was associated with improved outcomes (Figures 6(a) and 6(b)). As HRD positivity is increasingly being used as a means to guide the use of neoadjuvant chemotherapy, PARPi, or other targeted therapy across multiple cancer types, our meta-analysis suggests prognostic differences in these patients with HRD positivity, which may be targetable to improve outcomes [2, 19].

While the present study has limitations of small numbers of publications in certain subgroups, a relatively large number of publications with *BRCA1/2*m in breast and ovarian cancers were identified but not in other cancer types. Additionally, there were insufficient data points for HRR genes to perform a meta-analysis for each gene and group genes together in the summary. Furthermore, the methods used to identify HRRm and HRD in tumors vary by study and continue to evolve, and assays utilizing RNA or methylation techniques were excluded. Due to the limited number of published studies and inconsistent methodology and definitions of HRRm and HRD positivity, these results should be interpreted with caution.

It is puzzling that the results are not in consistent directions. This is the value of this comprehensive review that we brought to the literature. It is the reason that the treatments have to be tested in each of the cancers, their subtypes, and their mutation profiles. We have added this in the text. In addition, different studies could use different lists to determine pathogenic or VUS for BRCA mutations. There are some common rules: truncation mutations (stop gained, frameshift, and splice site), homozygous copy number deletions, and functional rearrangements. However, for those missense mutations, due to database curation and updating, the list to determine pathogenic or VUS could be different. Studies typically do not report such details in their publications. Given this is a literature review, we rely on the original papers regarding the definition of pathogenic and VUS mutations, as one of the limitations of the literature review.

In conclusion, this systematic review and meta-analysis evaluated the prognostic value of *BRCA1/2* and HRR pathway gene mutations and HRD positivity in multiple cancers. These findings should prove useful in improving the precision and efficacy of treatment selection in clinical oncology. Given the significantly improved outcomes following treatment with PARPi and augmented synthetic lethality to platinum agents in patients with tumors that have markers of HRD, the development and standardization of biomarker assays could have important clinical implications in discovering novel synthetic lethal combination therapies to improve outcomes for cancer patients.

## Data Availability

The data used to support the findings of this study are publicly available and listed in the supplementary material of this article.

## Supplementary Materials

Supplementary Materials Supplementary Table 1. Eligibility criteria for study inclusion. Supplementary Table 2. Articles included on BRCA and overall survival. Supplementary Table 3. Articles included on HRR and overall survival. Supplementary Table 4. Articles included on HRD and overall survival. Supplementary File 5: Supplementary Figure 1(a). BRCA1 and BRCA2: a meta-analysis of OS among breast cancer patients with germline tumor testing only. Supplementary Figure 1(b). BRCA1 and BRCA2: a meta-analysis of OS among breast cancer patients with pathogenicity annotation/classification. Supplementary Figure 1(c). BRCA1 and BRCA2: a meta-analysis of OS among triple-negative breast cancer (TNBC) patients with germline tumor testing only. Supplementary Figure 1(d). BRCA1 and BRCA2: a meta-analysis of OS among triple-negative breast cancer (TNBC) patients with pathogenicity annotation/classification. Supplementary Figure 1(e). BRCA1 and BRCA2: a meta-analysis of OS among ovarian cancer patients with germline mutations only. Supplementary Figure 1(f). BRCA1 and BRCA2: a meta-analysis of OS among ovarian cancer patients with somatic mutations only. Supplementary Figure 1(g). BRCA 1 and BRCA2: a meta-analysis of OS among ovarian cancer patients with stage III-IV. Supplementary Figure 2(a). BRCA1 only: a meta-analysis of OS among breast cancer patients stratified by germline or somatic tumor testing. Supplementary Figure 2(b). BRCA1 only: a meta-analysis of OS among breast cancer patients with germline tumor testing only. Supplementary Figure 2(c). BRCA1 only: a meta-analysis of OS among breast cancer patients with pathogenicity annotation/classification. Supplementary Figure 2(d). BRCA1 only: a meta-analysis of OS among triple-negative breast cancer (TNBC) patients. Supplementary Figure 2(e). BRCA1 only: a meta-analysis of OS among ovarian cancer patients with germline mutations only. Supplementary Figure 2(f). BRCA1 only: a meta-analysis of OS among ovarian cancer patients with combined information on both germline and somatic mutations. Supplementary Figure 2(g). BRCA1 only: a meta-analysis of OS among ovarian cancer patients with pathogenicity annotation/classification. Supplementary Figure 2(h). BRCA1 only: a meta-analysis of OS among ovarian cancer patients with stage III-IV. Supplementary Figure 3(a). BRCA2 only: a meta-analysis of OS among breast cancer patients with germline tumor testing only. Supplementary Figure 3(b). BRCA2 only: a meta-analysis of OS among breast cancer patients with pathogenicity annotation/classification. Supplementary Figure 3(c). BRCA2 only: a meta-analysis of OS among ovarian cancer patients with germline mutations only. Supplementary Figure 3(d). BRCA2 only: a meta-analysis of OS among ovarian cancer patients with combined information on both germline and somatic mutations. Supplementary Figure 3(e). BRCA2 only: a meta-analysis of OS among ovarian cancer patients with pathogenicity annotation/classification. Supplementary Figure 3(f). BRCA2 only: a meta-analysis of OS among ovarian cancer patients with pathogenicity annotation/classification. Supplementary Figure 3(g). BRCA2 only: a meta-analysis of OS among ovarian cancer patients with stage III-IV. Supplementary Figure 3(h). BRCA2 only: a meta-analysis of OS among prostate cancer patients with information on both germline and/or somatic testing. Supplementary Figure 3(i). BRCA2 only: a meta-analysis of OS among prostate cancer patients with information on germline testing. Supplementary Figure 3(j). BRCA2 only: a meta-analysis of OS among prostate cancer patients with pathogenicity annotation/classification. Supplementary Figure 4(a). HRR (ATM gene): a meta-analysis of OS among all patients. Supplementary Figure 4(b). HRR (two genes): a meta-analysis of OS among all patients. Supplementary Figure 4(c). HRR (three or more genes): a meta-analysis of OS among all patients.
