# Supplementary material for: A Systematic Review and Meta-Analysis on the Prognostic Value of BRCA Mutations, Homologous Recombination Gene Mutations, and Homologous Recombination Deficiencies in Cancer
Source: J Oncol. 2022 Jul 20;2022:5830475. doi: 10.1155/2022/5830475 (PMC9328957; doi:10.1155/2022/5830475)
Supplement: Supplementary Materials — Supplementary Table 1. Eligibility criteria for study inclusion. Supplementary Table 2. Articles included on BRCA and overall survival. Supplementary Table 3. Articles included on HRR and overall survival. Supplementary Table 4. Articles included on HRD and overall survival. Supplementary File 5: Supplementary Figure 1(a). BRCA1 and BRCA2: a meta-analysis of OS among breast cancer patients with germline tumor testing only. Supplementary Figure 1(b). BRCA1 and BRCA2: a meta-analysis of OS among breast cancer patients with pathogenicity annotation/classification. Supplementary Figure 1(c). BRCA1 and BRCA2: a meta-analysis of OS among triple-negative breast cancer (TNBC) patients with germline tumor testing only. Supplementary Figure 1(d). BRCA1 and BRCA2: a meta-analysis of OS among triple-negative breast cancer (TNBC) patients with pathogenicity annotation/classification. Supplementary Figure 1(e). BRCA1 and BRCA2: a meta-analysis of OS among ovarian cancer patients with germline mutations only. Supplementary Figure 1(f). BRCA1 and BRCA2: a meta-analysis of OS among ovarian cancer patients with somatic mutations only. Supplementary Figure 1(g). BRCA 1 and BRCA2: a meta-analysis of OS among ovarian cancer patients with stage III-IV. Supplementary Figure 2(a). BRCA1 only: a meta-analysis of OS among breast cancer patients stratified by germline or somatic tumor testing. Supplementary Figure 2(b). BRCA1 only: a meta-analysis of OS among breast cancer patients with germline tumor testing only. Supplementary Figure 2(c). BRCA1 only: a meta-analysis of OS among breast cancer patients with pathogenicity annotation/classification. Supplementary Figure 2(d). BRCA1 only: a meta-analysis of OS among triple-negative breast cancer (TNBC) patients. Supplementary Figure 2(e). BRCA1 only: a meta-analysis of OS among ovarian cancer patients with germline mutations only. Supplementary Figure 2(f). BRCA1 only: a meta-analysis of OS among ovarian cancer patients with combined informatio [file 5830475.f1.zip › 5830475.f1/description.of.supplementaryfiles.docx]

**Supplementary File 1:**

**Supplementary Table 1. Eligibility criteria for study inclusion**

**Supplementary File 2:**

**Supplementary Table 2. Articles included on BRCA and overall survival**

**Supplementary File 3:**

**Supplementary Table 3. Articles included on HRR and overall survival**

**Supplementary File 4:**

**Supplementary Table 4. Articles included on HRD and overall survival**

**Supplementary File 5: Supplementary Figures**

**Supplementary Figure 1A**. BRCA1 and BRCA2: a meta-analysis of OS among breast cancer patients with germline tumor testing only

**Supplementary Figure 1B**. BRCA1 and BRCA2: a meta-analysis of OS among breast cancer patients with pathogenicity annotation/classification

**Supplementary Figure 1C.** BRCA1 and BRCA2: a meta-analysis of OS among triple negative breast cancer (TNBC) patients with germline tumor testing only

**Supplementary Figure 1D.** BRCA1 and BRCA2: a meta-analysis of OS among triple negative breast cancer (TNBC) patients with pathogenicity annotation/classification

**Supplementary Figure 1E**. BRCA1 and BRCA2: a meta-analysis of OS among ovarian cancer patients with germline mutations only

**Supplementary Figure 1F.** BRCA1 and BRCA2: a meta-analysis of OS among ovarian cancer patients with somatic mutations only

**Supplementary Figure 1G.** BRCA 1 and BRCA2: a meta-analysis of OS among ovarian cancer patients with stage III-IV

**Supplementary Figure 2A.** BRCA1 only: a meta-analysis of OS among breast cancer patients stratified by germline or somatic tumor testing

**Supplementary Figure 2B.** BRCA1 only: a meta-analysis of OS among breast cancer patients with germline tumor testing only

**Supplementary Figure 2C.** BRCA1 only: a meta-analysis of OS among breast cancer patients with pathogenicity annotation/classification

**Supplementary Figure 2D.** BRCA1 only: a meta-analysis of OS among triple negative breast cancer (TNBC) patients

**Supplementary Figure 2E.** BRCA1 only: a meta-analysis of OS among ovarian cancer patients with germline mutations only

**Supplementary Figure 2F.** BRCA1 only: a meta-analysis of OS among ovarian cancer patients with combined information on both germline and somatic mutations

**Supplementary Figure 2G.** BRCA1 only: a meta-analysis of OS among ovarian cancer patients with pathogenicity annotation/classification

**Supplementary Figure 2H.** BRCA1 only: a meta-analysis of OS among ovarian cancer patients with stage III-IV

**Supplementary Figure 3A**. BRCA2 only: a meta-analysis of OS among breast cancer patients with germline tumor testing only

**Supplementary Figure 3B.** BRCA2 only: a meta-analysis of OS among breast cancer patients with pathogenicity annotation/classification

**Supplementary Figure 3C.** BRCA2 only: a meta-analysis of OS among ovarian cancer patients with germline mutations only

**Supplementary Figure 3D.** BRCA2 only: a meta-analysis of OS among ovarian cancer patients with combined information on both germline and somatic mutations

**Supplementary Figure 3E.** BRCA2 only: a meta-analysis of OS among ovarian cancer patients with pathogenicity annotation/classification

**Supplementary Figure 3F.** BRCA2 only: a meta-analysis of OS among ovarian cancer patients with pathogenicity annotation/classification

**Supplementary Figure 3G.** BRCA2 only: a meta-analysis of OS among ovarian cancer patients with stage III-IV

**Supplementary Figure 3H.** BRCA2 only: a meta-analysis of OS among prostate cancer patients with information on both germline and/or somatic testing

**Supplementary Figure 3I.** BRCA2 only: a meta-analysis of OS among prostate cancer patients with information on germline testing

**Supplementary Figure 3J.** BRCA2 only: a meta-analysis of OS among prostate cancer patients with pathogenicity annotation/classification

**Supplementary Figure 4A**. HRR (ATM Gene): a meta-analysis of OS among all patients

**Supplementary Figure 4B.** HRR (Two Genes): a meta-analysis of OS among all patients

**Supplementary Figure 4C.** HRR (Three or More Genes): a meta-analysis of OS among all patients
