# Supplementary material for: A Systematic Review and Meta-Analysis on the Prognostic Value of BRCA Mutations, Homologous Recombination Gene Mutations, and Homologous Recombination Deficiencies in Cancer
Source: J Oncol. 2022 Jul 20;2022:5830475. doi: 10.1155/2022/5830475 (PMC9328957; doi:10.1155/2022/5830475)
Supplement: Supplementary Materials — Supplementary Table 1. Eligibility criteria for study inclusion. Supplementary Table 2. Articles included on BRCA and overall survival. Supplementary Table 3. Articles included on HRR and overall survival. Supplementary Table 4. Articles included on HRD and overall survival. Supplementary File 5: Supplementary Figure 1(a). BRCA1 and BRCA2: a meta-analysis of OS among breast cancer patients with germline tumor testing only. Supplementary Figure 1(b). BRCA1 and BRCA2: a meta-analysis of OS among breast cancer patients with pathogenicity annotation/classification. Supplementary Figure 1(c). BRCA1 and BRCA2: a meta-analysis of OS among triple-negative breast cancer (TNBC) patients with germline tumor testing only. Supplementary Figure 1(d). BRCA1 and BRCA2: a meta-analysis of OS among triple-negative breast cancer (TNBC) patients with pathogenicity annotation/classification. Supplementary Figure 1(e). BRCA1 and BRCA2: a meta-analysis of OS among ovarian cancer patients with germline mutations only. Supplementary Figure 1(f). BRCA1 and BRCA2: a meta-analysis of OS among ovarian cancer patients with somatic mutations only. Supplementary Figure 1(g). BRCA 1 and BRCA2: a meta-analysis of OS among ovarian cancer patients with stage III-IV. Supplementary Figure 2(a). BRCA1 only: a meta-analysis of OS among breast cancer patients stratified by germline or somatic tumor testing. Supplementary Figure 2(b). BRCA1 only: a meta-analysis of OS among breast cancer patients with germline tumor testing only. Supplementary Figure 2(c). BRCA1 only: a meta-analysis of OS among breast cancer patients with pathogenicity annotation/classification. Supplementary Figure 2(d). BRCA1 only: a meta-analysis of OS among triple-negative breast cancer (TNBC) patients. Supplementary Figure 2(e). BRCA1 only: a meta-analysis of OS among ovarian cancer patients with germline mutations only. Supplementary Figure 2(f). BRCA1 only: a meta-analysis of OS among ovarian cancer patients with combined informatio [file 5830475.f1.zip › 5830475.f1/Supplementaryfile1.Supplementary Tables 1.docx]

**Supplementary Table 1. Eligibility criteria for study inclusion**

| Criteria | Description |
| --- | --- |
| Population | - Patients with solid tumors - Cancer patients tested for either *BRCA*1m or *BRCA2m* or - Cancer patients tested for HRR mutations or - Cancer patients tested for HRD |
| Outcomes | - Clinical outcomes were described by tumor type, histologic subtype, by stage, by region/country, by gender if data were available   Specifically,   - Clinical outcomes (e.g., OS, PFS, ORR) for BRCAm and BRCA wide type (BRCAwt), by tumor type and subtype - Clinical outcomes (e.g., OS, PFS, ORR) for HRRm and HRRwt, by tumor type and subtype - Clinical outcomes (e.g., OS, PFS, ORR) for HRD+ and HRD-, by tumor type and subtype |
| Study design | Both observational studies and randomized as well as single arm clinical trial data that were in the scope if data were available.   - Prospective and retrospective cohort studies - Case-control studies - Cross-sectional studies - Controlled and uncontrolled longitudinal studies (cohorts or case series) |
| Language | - Only studies published in English were included |
| Time | - Literature published in the past 10 years. - Conference abstracts in the past 3 years. |
