# Supplementary material for: A Systematic Review and Meta-Analysis on the Prognostic Value of BRCA Mutations, Homologous Recombination Gene Mutations, and Homologous Recombination Deficiencies in Cancer
Source: J Oncol. 2022 Jul 20;2022:5830475. doi: 10.1155/2022/5830475 (PMC9328957; doi:10.1155/2022/5830475)
Supplement: Supplementary Materials — Supplementary Table 1. Eligibility criteria for study inclusion. Supplementary Table 2. Articles included on BRCA and overall survival. Supplementary Table 3. Articles included on HRR and overall survival. Supplementary Table 4. Articles included on HRD and overall survival. Supplementary File 5: Supplementary Figure 1(a). BRCA1 and BRCA2: a meta-analysis of OS among breast cancer patients with germline tumor testing only. Supplementary Figure 1(b). BRCA1 and BRCA2: a meta-analysis of OS among breast cancer patients with pathogenicity annotation/classification. Supplementary Figure 1(c). BRCA1 and BRCA2: a meta-analysis of OS among triple-negative breast cancer (TNBC) patients with germline tumor testing only. Supplementary Figure 1(d). BRCA1 and BRCA2: a meta-analysis of OS among triple-negative breast cancer (TNBC) patients with pathogenicity annotation/classification. Supplementary Figure 1(e). BRCA1 and BRCA2: a meta-analysis of OS among ovarian cancer patients with germline mutations only. Supplementary Figure 1(f). BRCA1 and BRCA2: a meta-analysis of OS among ovarian cancer patients with somatic mutations only. Supplementary Figure 1(g). BRCA 1 and BRCA2: a meta-analysis of OS among ovarian cancer patients with stage III-IV. Supplementary Figure 2(a). BRCA1 only: a meta-analysis of OS among breast cancer patients stratified by germline or somatic tumor testing. Supplementary Figure 2(b). BRCA1 only: a meta-analysis of OS among breast cancer patients with germline tumor testing only. Supplementary Figure 2(c). BRCA1 only: a meta-analysis of OS among breast cancer patients with pathogenicity annotation/classification. Supplementary Figure 2(d). BRCA1 only: a meta-analysis of OS among triple-negative breast cancer (TNBC) patients. Supplementary Figure 2(e). BRCA1 only: a meta-analysis of OS among ovarian cancer patients with germline mutations only. Supplementary Figure 2(f). BRCA1 only: a meta-analysis of OS among ovarian cancer patients with combined informatio [file 5830475.f1.zip › 5830475.f1/Supplementaryfile2.Supplementary Table 2.BRCA-OS-outcome.pdf]

Articles included on BRCA and overall survial

Type	First author, Year	Journal	Title	Volume	Issue	Pages	Figure Number
abstract	CORTESI 2010	BMC CANCER	FAVOURABLE TEN-YEAR OVERALL SURVIVAL IN A CAUCASIAN POPULATION WITH HIGH PROBABILITY OF HEREDITARY BREAST CANCER	10	90	1	3A
abstract	CUNNINGHAM 2014	SCI REP	CLINICAL CHARACTERISTICS OF OVARIAN CANCER CLASSIFIED BY BRCA1, BRCA2, AND RAD51C STATUS	4	-	4026	3B, 4B
abstract	DANN 2012	GYNECOL ONCOL	BRCA1/2 MUTATIONS AND EXPRESSION: RESPONSE TO PLATINUM CHEMOTHERAPY IN PATIENTS WITH ADVANCED STAGE EPITHELIAL OVARIAN CANCER	125	3	677-82	2B
abstract	EDWARDS 2010	BR J CANCER	PROSTATE CANCER IN BRCA2 GERMLINE MUTATION CARRIERS IS ASSOCIATED WITH POORER PROGNOSIS	103	6	918-924	4C
abstract	NORQUIST 2016	JAMA ONCOL	INHERITED MUTATIONS IN WOMEN WITH OVARIAN CARCINOMA	2	4	482-90	3B, 4B
abstract	ECCLES 2018	BREAST CANCER RESEARCH AND TREATMENT	IMPACT OF A BRCA GERMLINE MUTATION ON SURVIVAL-PROSPECTIVE STUDY OF OUTCOMES IN SPORADIC VERSUS HEREDITARY BREAST CANCER (POSH)	19	2	169-180	2A
abstract	LIU 2019	JOURNAL OF CLINICAL ONCOLOGY	RESPONSE TO IMMUNE CHECKPOINT INHIBITION AND SURVIVAL IN BRCA-ASSOCIATED RECURRENT OVARIAN CANCER	37	15 suppl	-	2B
abstract	TYULYANDINA 2019	JOURNAL OF CLINICAL ONCOLOGY	IMPACT OF BRCA MUTATION STATUS AND TIME TO PLATINUM RESISTANCE ON PATIENTS WITH ADVANCED OVARIAN CANCER	37	15 suppl	-	2B
abstract	VLACHOSTERGIOS 2019	CANCER RESEARCH	PROGNOSTIC VALUE OF BRCA2 AND AR GENE ALTERATIONS IN ADVANCED PROSTATE CANCER PATIENTS TREATED WITH PSMA-TARGETED RADIONUCLIDE THERAPIES	79	13 supplement	-	4C
abstract	ZANG 2019	JOURNAL OF CLINICAL ONCOLOGY	DOSE-DENSE EARLY POSTOPERATIVE INTRAPERITONEAL CHEMOTHERAPY IN OVARIAN CANCER: RANDOMIZED, PHASE II TRIAL	37	15 suppl	-	2B
paper	ALSOP 2012	JCO	BRCA MUTATION FREQUENCY AND PATTERNS OF TREATMENT RESPONSE IN BRCA MUTATION-POSITIVE WOMEN WITH OVARIAN CANCER: A REPORT FROM THE AUSTRALIAN OVARIAN CANCER STUDY GROUP	30	21	2654-2663	2B, 3B, 4B
paper	ARPINO 2016	BMC CANCER	TUMOR CHARACTERISTICS AND PROGNOSIS IN FAMILIAL BREAST CANCER	16	1		2A
paper	BAYRAKTAR 2013	CLIN EXP METASTASIS	OUTCOME OF METASTATIC BREAST CANCER IN SELECTED WOMEN WITH OR WITHOUT DELETERIOUS BRCA1 AND BRCA2 MUTATIONS	30	5	631-642	3A, 4A
paper	BLAIR 2018	J AM COLL SURG	BRCA1/BRCA2 GERMLINE MUTATION CARRIERS AND SPORADIC PANCREATIC DUCTAL ADENOCARCINOMA	226	4	630-637	2C
paper	BOOKMAN 2017	GYNECOLOGIC ONCOLOGY	IMPACT OF PRIMARY PLATINUM-FREE INTERVAL AND BRCA1/2 MUTATION STATUS ON TREATMENT AND SURVIVAL IN PATIENTS WITH RECURRENT OVARIAN CANCER	146	1	58-63	2B

paper	BOUDIN 2016	BONE MARROW TRANSPLANT	HIGHLY FAVORABLE OUTCOME IN BRCA-MUTATED METASTATIC BREAST CANCER PATIENTS RECEIVING HIGH-DOSE CHEMOTHERAPY AND AUTOLOGOUS HEMATOPOIETIC STEM CELL TRANSPLANTATION	51	8	1082-1086	2A
paper	BU 2019	JOURNAL OF OBSTETRICS AND GYNAECOLOGY RESEARCH	BRCA MUTATION FREQUENCY AND CLINICAL FEATURES OF OVARIAN CANCER PATIENTS: A REPORT FROM A CHINESE STUDY GROUP	45	11	2267-2274	2B
paper	CASTRO 2013	J CLIN ONCOL	GERMLINE BRCA1 AND BRCA2 MUTATIONS ARE ASSOCIATED WITH HIGHER RISK OF NODAL INVOLVEMENT, DISTANT METASTASIS, AND POOR SURVIVAL OUTCOMES IN PROSTATE CANCER.	31	14	1748-1757	4C
paper	CECENER 2020	CANCER GENETICS	CLINICOPATHOLOGIC FEATURES AND GENETIC CHARACTERISTICS OF THE BRCA1/2 MUTATION IN TURKISH BREAST CANCER PATIENTS	240		23-32	3A, 4A
paper	CLIFTON 2018	BREAST CANCER RESEARCH AND TREATMENT	ADJUVANT VERSUS NEOADJUVANT CHEMOTHERAPY IN TRIPLE-NEGATIVE BREAST CANCER PATIENTS WITH BRCA1 AND BRCA2 MUTATIONS	170	1	101-109	2A
paper	COPSON 2018	LANCET ONCOLOGY	GERMLINE BRCA MUTATION AND OUTCOME IN YOUNG-ONSET BREAST CANCER (POSH): A PROSPECTIVE COHORT STUDY	19	2	169-180	2A, 3A, 4A
paper	CRONIN-FENTON 2017	CANCER EPIDEMIOLOG Y	CLINICAL OUTCOMES OF FEMALE BREAST CANCER ACCORDING TO BRCA MUTATION STATUS	49		128-137	3A
paper	DENG 2019	INTERNATIONA L JOURNAL OF CANCER	PREVALENCE AND CLINICAL OUTCOMES OF GERMLINE MUTATIONS IN BRCA1/2 AND PALB2 GENES IN 2769 UNSELECTED BREAST CANCER PATIENTS IN CHINA	145	6	1517-1528	2A, 3A, 4A
paper	DIMITROVA 2016	TUMOR BIOLOGY	GERMLINE MUTATIONS OF BRCA1 GENE EXON 11 ARE NOT ASSOCIATED WITH PLATINUM RESPONSE NEITHER WITH SURVIVAL ADVANTAGE IN PATIENTS WITH PRIMARY OVARIAN CANCER: UNDERSTANDING THE CLINICAL IMPORTANCE OF ONE OF THE BIGGEST HUMAN EXONS. A STUDY OF THE TUMOR BANK OVARIAN	37	9	12329-12337	3B
paper	DONG 2016	CANCER EPIDEMIOLOG Y BIOMARKERS AND PREVENTION	A BRCA1/2 MUTATIONAL SIGNATURE AND SURVIVAL IN OVARIAN HIGH-GRADE SEROUS CARCINOMA	25	11	1511-1516	2B
paper	ELSAKOV 2016	BREAST CANCER MANAGEMENT	MANAGEMENT AND 5-YEAR SURVIVAL OF BRCA1-ASSOCIATED BREAST CANCER PATIENTS	5	3	119-124	2A
paper	EOH 2020	BMC CANCER	MUTATION LANDSCAPE OF GERMLINE AND SOMATIC BRCA1/2 IN PATIENTS WITH HIGH-GRADE SEROUS OVARIAN CANCER	20	1		2B
paper	EOH 2017	CANCER RESEARCH AND TREATMENT	COMPARISON OF CLINICAL OUTCOMES OF BRCA1/2 PATHOLOGIC MUTATION, VARIANTS OF UNKNOWN SIGNIFICANCE, OR WILD TYPE EPITHELIAL OVARIAN CANCER PATIENTS	49	2	408-415	2B

paper	GALLAGHER 2011	ANNALS OF ONCOLOGY	SURVIVAL IN EPITHELIAL OVARIAN CANCER: A MULTIVARIATE ANALYSIS INCORPORATING BRCA MUTATION STATUS AND PLATINUM SENSITIVITY	22	5	1127-1132	2B
paper	GOLAN 2017	BRITISH JOURNAL OF CANCER	OVERALL SURVIVAL AND CLINICAL CHARACTERISTICS OF BRCA MUTATION CARRIERS WITH STAGE I/II PANCREATIC CANCER	116	6	694-702	2C
paper	GONZALEZ-ANGULO 2011	CLIN CANCER RES	INCIDENCE AND OUTCOME OF BRCA1 AND BRCA2 MUTATIONS IN UNSELECTED PATIENTS WITH TRIPLE RECEPTOR-NEGATIVE BREAST CANCER	17	5	1082-1089	2A
paper	HYMAN 2012	GYNECOLOGIC ONCOLOGY	OUTCOMES OF PRIMARY SURGICAL CYTOREDUCTION IN PATIENTS WITH BRCA-ASSOCIATED HIGH-GRADE SEROUS OVARIAN CARCINOMA	126	2	224-228	3B, 4B
paper	KIM 2019	JOURNAL OF OVARIAN RESEARCH	EFFECT OF BRCA MUTATIONAL STATUS ON SURVIVAL OUTCOME IN ADVANCED-STAGE HIGH-GRADE SEROUS OVARIAN CANCER	12	1		2B
paper	KIROVA 2010	BREAST CANCER RES TREAT	IS THE BREAST-CONSERVING TREATMENT WITH RADIOTHERAPY APPROPRIATE IN BRCA1/2 MUTATION CARRIERS? LONG-TERM RESULTS AND REVIEW OF THE LITERATURE	120	1	119-126	2A
paper	KOHLI 2020	EBIOMEDICINE	CLINICAL AND GENOMIC INSIGHTS INTO CIRCULATING TUMOR DNA-BASED ALTERATIONS ACROSS THE SPECTRUM OF METASTATIC HORMONE-SENSITIVE AND CASTRATE-RESISTANT PROSTATE CANCER	54			4C
paper	KOLETSA 2014	BMC CLINICAL PATHOLOGY	ALPHAB-CRYSTALLIN IS A MARKER OF AGGRESSIVE BREAST CANCER BEHAVIOR BUT DOES NOT INDEPENDENTLY PREDICT FOR PATIENT OUTCOME: A COMBINED ANALYSIS OF TWO RANDOMIZED STUDIES	14	1		3A
paper	KOTSOPOULOS 2016	GYNECOLOGIC ONCOLOGY	TEN-YEAR SURVIVAL AFTER EPITHELIAL OVARIAN CANCER IS NOT ASSOCIATED WITH BRCA MUTATION STATUS	140	1	42-47	3B
paper	MCLAUGHLIN 2013	JOURNAL OF THE NATIONAL CANCER INSTITUTE	LONG-TERM OVARIAN CANCER SURVIVAL ASSOCIATED WITH MUTATION IN BRCA1 OR BRCA2	105	2	141-148	2B, 3B, 4B
paper	MEISEL 2014	ANNALS OF ONCOLOGY	THE PERFORMANCE OF BRCA1 IMMUNOHISTOCHEMISTRY FOR DETECTING GERMLINE, SOMATIC, AND EPIGENETIC BRCA1 LOSS IN HIGH-GRADE SEROUS OVARIAN CANCER	25	12	2372-2378	3B
paper	NIENTIEDT 2017	SCIENTIFIC REPORTS	MUTATIONS IN BRCA2 AND TAXANE RESISTANCE IN PROSTATE CANCER	7	1	4574	4C
paper	NILSSON 2014	BREAST CANCER RES TREAT	LONG-TERM PROGNOSIS OF EARLY-ONSET BREAST CANCER IN A POPULATION-BASED COHORT WITH A KNOWN BRCA1/2 MUTATION STATUS	144	1	133-142	2A
paper	PALUCH-SHIMON 2016	BREAST CANCER RESEARCH AND TREATMENT	NEO-ADJUVANT DOXORUBICIN AND CYCLOPHOSPHAMIDE FOLLOWED BY PACLITAXEL IN TRIPLE-NEGATIVE BREAST CANCER AMONG BRCA1 MUTATION CARRIERS AND NON-CARRIERS	157	1	157-165	3A
paper	POGODA 2020	JOURNAL OF ONCOLOGY	EFFECTS OF BRCA GERMLINE MUTATIONS ON TRIPLE-NEGATIVE BREAST CANCER PROGNOSIS	2020			2A
paper	POP 2018	BREAST	GENETIC ALTERATIONS IN SPORADIC TRIPLE NEGATIVE BREAST CANCER	38		30-38	3A, 4A

paper	RUDAITIS 2014	INTERNATIONAL JOURNAL OF GYNECOLOGICAL CANCER	BRCA1/2 MUTATION STATUS IS AN INDEPENDENT FACTOR OF IMPROVED SURVIVAL FOR ADVANCED (STAGE III-IV) OVARIAN CANCER	24	8	1395-1400	2B
paper	RYU 2018	BREAST CANCER RESEARCH AND TREATMENT	PREVALENCE AND ONCOLOGIC OUTCOMES OF BRCA 1/2 MUTATIONS IN UNSELECTED TRIPLE-NEGATIVE BREAST CANCER PATIENTS IN KOREA	173	2	385-395	2A
paper	RZEPECKA 2016	GYNECOLOGIC ONCOLOGY	PROGNOSIS OF PATIENTS WITH BRCA1-ASSOCIATED OVARIAN CARCINOMAS DEPENDS ON TP53 ACCUMULATION STATUS IN TUMOR CELLS	144	2	369-376	3B
paper	SABATIER 2016	FAMILIAL CANCER	OVARIAN CANCER PATIENTS AT HIGH RISK OF BRCA MUTATION: THE CONSTITUTIONAL GENETIC CHARACTERIZATION DOES NOT CHANGE PROGNOSIS	15	4	497-506	2B
paper	SAFRA 2011	MOLECULAR CANCER THERAPEUTICS	BRCA MUTATION STATUS AND DETERMINANT OF OUTCOME IN WOMEN WITH RECURRENT EPITHELIAL OVARIAN CANCER TREATED WITH PEGYLATED LIPOSOMAL DOXORUBICIN	10	10	2000-2007	2B
paper	SAFRA 2013	ANNALS OF ONCOLOGY	BRCA1 AND BRCA2 MUTATIONS AND OUTCOME IN EPITHELIAL OVARIAN CANCER (EOC): EXPERIENCE IN ETHNICALLY DIVERSE GROUPS	24	SUPPL.8	VIII63-VIII68	2B, 3B, 4B
paper	SAFRA 2014	INTERNATIONAL JOURNAL OF GYNECOLOGICAL CANCER	THE EFFECT OF GERM-LINE BRCA1 AND BRCA2 MUTATIONS ON RESPONSE TO CHEMOTHERAPY AND OUTCOME OF RECURRENT OVARIAN CANCER	24	3	488-495	2B
paper	SCHMIDT 2017	JOURNAL OF THE NATIONAL CANCER INSTITUTE	BREAST CANCER SURVIVAL OF BRCA1/BRCA2 MUTATION CARRIERS IN A HOSPITAL-BASED COHORT OF YOUNG WOMEN	109	8		3A, 4A
paper	SHI 2018	CELLULAR PHYSIOLOGY AND BIOCHEMISTRY	SURVIVAL BENEFIT OF GERMLINE BRCA MUTATION IS ASSOCIATED WITH RESIDUAL DISEASE IN OVARIAN CANCER	47	5	2088-2096	2B
paper	STASENKO 2019	GYNECOLOGIC ONCOLOGY	BRAIN METASTASIS IN EPITHELIAL OVARIAN CANCER BY BRCA1/2 MUTATION STATUS	154	1	144-149	2A
paper	SUGINO 2019	SCIENTIFIC REPORTS	GERMLINE AND SOMATIC MUTATIONS OF HOMOLOGOUS RECOMBINATION-ASSOCIATED GENES IN JAPANESE OVARIAN CANCER PATIENTS	9	1	17808	2B
paper	THORNE 2011	CANCER PREVENTION RESEARCH	DECREASED PROSTATE CANCER-SPECIFIC SURVIVAL OF MEN WITH BRCA2 MUTATIONS FROM MULTIPLE BREAST CANCER FAMILIES	4	7	1002-1010	4C
paper	VENERIS 2019	INTERNATIONAL JOURNAL OF GYNECOLOGICAL CANCER	GLUCOCORTICOID RECEPTOR EXPRESSION IS ASSOCIATED WITH INFERIOR OVERALL SURVIVAL INDEPENDENT OF BRCA MUTATION STATUS IN OVARIAN CANCER	29	2	357-364	2B
paper	WANG 2018	BMC CANCER	GERMLINE BREAST CANCER SUSCEPTIBILITY GENE MUTATIONS AND BREAST CANCER OUTCOMES	18	1		2A
paper	YADAV 2017	CLINICAL BREAST CANCER	IMPACT OF BRCA MUTATION STATUS ON SURVIVAL OF WOMEN WITH TRIPLE-NEGATIVE BREAST CANCER	18	5	E1229-E1235	2A

paper	YANG 2011	JAMA	ASSOCIATION OF BRCA1 AND BRCA2 MUTATIONS WITH SURVIVAL, CHEMOTHERAPY SENSITIVITY, AND GENE MUTATOR PHENOTYPE IN PATIENTS WITH OVARIAN CANCER	306	14	1557-1565	3B, 4B
paper	YE 2020	CANCER MEDICINE	OUTCOMES AND RISK OF SUBSEQUENT BREAST EVENTS IN BREAST-CONSERVING SURGERY PATIENTS WITH BRCA1 AND BRCA2 MUTATION	9	5	1903-1910	2A
paper	YOU 2020	FRONTIERS IN ONCOLOGY	GERMLINE AND SOMATIC BRCA1/2 MUTATIONS IN 172 CHINESE WOMEN WITH EPITHELIAL OVARIAN CANCER	10			2B, 3B, 4B
paper	BAYRAKTAR 2011	BREAST CANCER RES TREAT	OUTCOME OF TRIPLE-NEGATIVE BREAST CANCER IN PATIENTS WITH OR WITHOUT DELETERIOUS BRCA1 AND BRCA2 MUTATIONS	130	1	145-153	2A
paper	GOODWIN 2011	J CLIN ONCOL	BREAST CANCER PROGNOSIS IN BRCA1 AND BRCA2 MUTATION CARRIERS: AN INTERNATIONAL PROSPECTIVE BREAST CANCER FAMILY REGISTRY POPULATION-BASED COHORT STUDY	30	1	19-26	3A, 4A
