# Supplementary material for: A Systematic Review and Meta-Analysis on the Prognostic Value of BRCA Mutations, Homologous Recombination Gene Mutations, and Homologous Recombination Deficiencies in Cancer
Source: J Oncol. 2022 Jul 20;2022:5830475. doi: 10.1155/2022/5830475 (PMC9328957; doi:10.1155/2022/5830475)
Supplement: Supplementary Materials — Supplementary Table 1. Eligibility criteria for study inclusion. Supplementary Table 2. Articles included on BRCA and overall survival. Supplementary Table 3. Articles included on HRR and overall survival. Supplementary Table 4. Articles included on HRD and overall survival. Supplementary File 5: Supplementary Figure 1(a). BRCA1 and BRCA2: a meta-analysis of OS among breast cancer patients with germline tumor testing only. Supplementary Figure 1(b). BRCA1 and BRCA2: a meta-analysis of OS among breast cancer patients with pathogenicity annotation/classification. Supplementary Figure 1(c). BRCA1 and BRCA2: a meta-analysis of OS among triple-negative breast cancer (TNBC) patients with germline tumor testing only. Supplementary Figure 1(d). BRCA1 and BRCA2: a meta-analysis of OS among triple-negative breast cancer (TNBC) patients with pathogenicity annotation/classification. Supplementary Figure 1(e). BRCA1 and BRCA2: a meta-analysis of OS among ovarian cancer patients with germline mutations only. Supplementary Figure 1(f). BRCA1 and BRCA2: a meta-analysis of OS among ovarian cancer patients with somatic mutations only. Supplementary Figure 1(g). BRCA 1 and BRCA2: a meta-analysis of OS among ovarian cancer patients with stage III-IV. Supplementary Figure 2(a). BRCA1 only: a meta-analysis of OS among breast cancer patients stratified by germline or somatic tumor testing. Supplementary Figure 2(b). BRCA1 only: a meta-analysis of OS among breast cancer patients with germline tumor testing only. Supplementary Figure 2(c). BRCA1 only: a meta-analysis of OS among breast cancer patients with pathogenicity annotation/classification. Supplementary Figure 2(d). BRCA1 only: a meta-analysis of OS among triple-negative breast cancer (TNBC) patients. Supplementary Figure 2(e). BRCA1 only: a meta-analysis of OS among ovarian cancer patients with germline mutations only. Supplementary Figure 2(f). BRCA1 only: a meta-analysis of OS among ovarian cancer patients with combined informatio [file 5830475.f1.zip › 5830475.f1/Supplementaryfile3.Supplementary Table 3.HRR-OS-outcome.pdf]

Articles included on HRR and overal survival

Type	First author	Title	Journal	Volume	Issue	Pages	Figure Number
abstract	Antonarakis 2018	Effect of germline DNA repair gene mutations on outcomes in men with metastatic castration-resistant prostate cancer receiving first-line abiraterone and enzalutamide.	JCO	36	6 suppl		5A, 5B
abstract	Fasching 2018	Cancer predisposition genes in metastatic breast cancer - Association with metastatic pattern, prognosis, patient and tumor characteristics	Cancer research	78	4 suppl		5A, 5B
abstract	Kasi 2018	Germline BRCA1/2, PALB2, and ATM mutations in 3,030 patients with pancreatic adenocarcinoma: Survival analysis of carriers and noncarriers	J Clin Oncol	36	4 suppl		5A, 5B
abstract	Van Der Doelen 2020	Overall survival using radium-223 (Ra223) in metastatic castrate-resistant prostate cancer (mCRPC) patients with and without DNA damage repair (DDR) defects	J Clin Oncol	38	6 suppl		5B
paper	Yin 2020	Prognostic Value of DNA Damage Response Genomic Alterations in Relapsed/Advanced Urothelial Cancer	Oncologist	25	8	680-688	5A, 5B
paper	Yu 2019	Retrospective survival analysis of patients with resected pancreatic ductal adenocarcinoma and a germline BRCA or PALB2 mutation	JCO Precision Oncology		3	1-11	5B
paper	Wu 2018	A comprehensive evaluation of CHEK2 germline mutations in men with prostate cancer	Prostate	78	8	607-615	5A, 5B
paper	Deng 2019	Prevalence and clinical outcomes of germline mutations in BRCA1/2 and PALB2 genes in 2769 unselected breast cancer patients in China	International Journal of Cancer	145	6	1517-1528	5A, 5B
paper	Kohli 2020	Clinical and genomic insights into circulating tumor DNA-based alterations across the spectrum of metastatic hormone-sensitive and castrate-resistant prostate cancer	EBioMedicine	54	2020	102728	5A, 5B
paper	Luo 2020	Clonal tumor mutations in homologous recombination genes predict favorable clinical outcome in ovarian cancer treated with platinum-based Chemotherapytherapy	Gynecologic Oncology	158	1	66-76	5A, 5B
paper	Na 2017	Germline Mutations in ATM and BRCA1/2 Distinguish Risk for Lethal and Indolent Prostate Cancer and are Associated with Early Age at Death	European Urology	71	5	740-747	5A, 5B
paper	Cunningham 2014	Clinical characteristics of ovarian cancer classified by BRCA1, BRCA2, and RAD51C status	Sci Rep	4	4026	1-7	5B
paper	Smith 2018	Reflex testing for germline BRCA1, BRCA2, PALB2, and ATM mutations in pancreatic cancer: Mutation prevalence and clinical outcomes from two Canadian research registries	JCO Precision Oncology	2			5A, 5B
paper	Sehdev 2018	Germline and somatic DNA damage repair gene mutations and overall survival in metastatic pancreatic adenocarcinoma patients treated with FOLFIRINOX	Clinical Cancer Research	24	24	6204-6211	5A, 5B
paper	Morse 2019	Tumor infiltrating lymphocytes and homologous recombination deficiency are independently associated with improved survival in ovarian carcinoma	Gynecologic Oncology	153	2	217-222	5A, 5B
