# Supplementary material for: A Systematic Review and Meta-Analysis on the Prognostic Value of BRCA Mutations, Homologous Recombination Gene Mutations, and Homologous Recombination Deficiencies in Cancer
Source: J Oncol. 2022 Jul 20;2022:5830475. doi: 10.1155/2022/5830475 (PMC9328957; doi:10.1155/2022/5830475)
Supplement: Supplementary Materials — Supplementary Table 1. Eligibility criteria for study inclusion. Supplementary Table 2. Articles included on BRCA and overall survival. Supplementary Table 3. Articles included on HRR and overall survival. Supplementary Table 4. Articles included on HRD and overall survival. Supplementary File 5: Supplementary Figure 1(a). BRCA1 and BRCA2: a meta-analysis of OS among breast cancer patients with germline tumor testing only. Supplementary Figure 1(b). BRCA1 and BRCA2: a meta-analysis of OS among breast cancer patients with pathogenicity annotation/classification. Supplementary Figure 1(c). BRCA1 and BRCA2: a meta-analysis of OS among triple-negative breast cancer (TNBC) patients with germline tumor testing only. Supplementary Figure 1(d). BRCA1 and BRCA2: a meta-analysis of OS among triple-negative breast cancer (TNBC) patients with pathogenicity annotation/classification. Supplementary Figure 1(e). BRCA1 and BRCA2: a meta-analysis of OS among ovarian cancer patients with germline mutations only. Supplementary Figure 1(f). BRCA1 and BRCA2: a meta-analysis of OS among ovarian cancer patients with somatic mutations only. Supplementary Figure 1(g). BRCA 1 and BRCA2: a meta-analysis of OS among ovarian cancer patients with stage III-IV. Supplementary Figure 2(a). BRCA1 only: a meta-analysis of OS among breast cancer patients stratified by germline or somatic tumor testing. Supplementary Figure 2(b). BRCA1 only: a meta-analysis of OS among breast cancer patients with germline tumor testing only. Supplementary Figure 2(c). BRCA1 only: a meta-analysis of OS among breast cancer patients with pathogenicity annotation/classification. Supplementary Figure 2(d). BRCA1 only: a meta-analysis of OS among triple-negative breast cancer (TNBC) patients. Supplementary Figure 2(e). BRCA1 only: a meta-analysis of OS among ovarian cancer patients with germline mutations only. Supplementary Figure 2(f). BRCA1 only: a meta-analysis of OS among ovarian cancer patients with combined informatio [file 5830475.f1.zip › 5830475.f1/Supplementaryfile4.Supplementary Table 4.HRD-OS-outcome.pdf]

Articles included on HRD and overal survival

Type	Author, year	Journal	Title	Volume	Issue	Pages
paper	LOIBL, 2017	ANNALS OF ONCOLOGY : OFFICIAL JOURNAL OF THE EUROPEAN SOCIETY FOR MEDICAL ONCOLOGY	SURVIVAL ANALYSIS OF CARBOPLATIN ADDED TO AN ANTHRACYCLINE/TAXANE-BASED NEOADJUVANT CHEMOTHERAPY AND HRD SCORE AS PREDICTOR OF RESPONSE-FINAL RESULTS FROM GEPARSIXTO	29	12	2341-2347
paper	SHAHDA 2018	JCO PRECISION ONCOLOGY	HOMOLOGOUS RECOMBINATION DEFICIENCY IN PATIENTS WITH PANCREATIC DUCTAL ADENOCARCINOMA AND RESPONSE TO CHEMOTHERAPY	2		1-11
paper	SHARMA 2018	ANNALS OF ONCOLOGY	IMPACT OF HOMOLOGOUS RECOMBINATION DEFICIENCY BIOMARKERS ON OUTCOMES IN PATIENTS WITH TRIPLENEGATIVE BREAST CANCER TREATED WITH ADJUVANT DOXORUBICIN AND CYCLOPHOSPHAMIDE (SWOG S9313)	29	3	654-660
paper	VANDERSTICHELE 2017	INTERNATIONAL JOURNAL OF GYNECOLOGICAL CANCER	CLINICAL CHARACTERISTICS AND GENOMIC BIOMARKERS OF THE HOMOLOGOUS RECOMBINATION DEFICIENCY PHENOTYPE IN HIGH-GRADE SEROUS OVARIAN CANCER.	86		5-14
paper	CUNNINGHAM 2014	SCI REP	CLINICAL CHARACTERISTICS OF OVARIAN CANCER CLASSIFIED BY BRCA1, BRCA2 AND RAD51C STATUS	4		4026
paper	TUMIATI 2018	CLINICAL CANCER RESEARCH	A FUNCTIONAL HOMOLOGOUS RECOMBINATION ASSAY PREDICTS PRIMARY CHEMOTHERAPY RESPONSE AND LONG-TERM SURVIVAL IN OVARIAN CANCER PATIENTS	24	18	4482-4493
abstract	SOUSA 2019	JCO	HOMOLOGOUS RECOMBINATION DEFICIENCY AS PROGNOSTIC MARKER IN METASTATIC GASTRIC CANCER	37	15_suppl	4040
